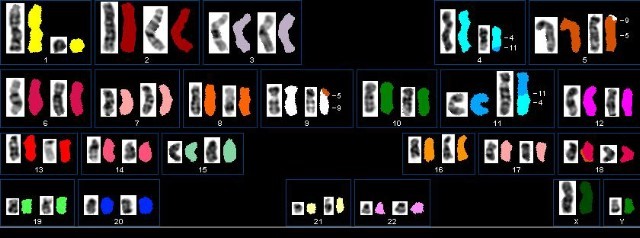# Spectral karyotyping (SKY)

**DOI:** 10.3332/ecancer.2010.181

**Published:** 2010-10-13

**Authors:** E Belloni, E Bonnomi, I Lahortiga, MD Odero, PP Di Fiore, PG Pelicci

**Affiliations:** 1 Istituto Europeo di Oncologia, Milan, Italy; 2 Campus IFOM-IEO, Milan, Italy; 3 Department of Genetics, University of Navarra, Pamplona, Spain; 4 Universita’ degli Studi di Milano, Milan, Italy

Spectral karyotyping (SKY) enables the visualization of all 24 human chromosomes assigned in different colours in a single metaphase, by using a combination of probe labelling, fluorescence microscopy, spectroscopy, CCD-imaging and spectral image analysis. The benefits of such an approach include the accurate analysis of abnormal karyotypes, unresolved by conventional cytogenetics and the ability to identify cryptic translocations in apparently ‘normal’ karyotypes. The labelling procedure is based on the utilization of five fluorochromes, differently combined for each specific chromosome. The SKY system (microscope and acquisition software) receives and processes the emitted signals, assigning a specific colour to a specific chromosome. In this image, the karyotype of a male affected by acute myeloid leukaemia is represented, showing the presence of the following rearrangements: del1q, balanced t(4;11) and balanced t(5;9). [Realized with the collaboration of Dr Idoya Lahortiga, visiting our institute in 2001.]

**Figure:**