# Erratum: Spectral Karyotyping (SKY)

**DOI:** 10.3332/ecancer.2012.256

**Published:** 2012-06-06

**E Belloni, E Bonnomi, I Lahortiga, MD Odero, PP Di Fiore, PG Pelicci**

*e*cancer **4** 181 (2010)

The name “E Bonnomi” was incorrectly added to the author list of this article.

